# Altered PGE2-EP2 is associated with an excessive immune response in HBV-related acute-on-chronic liver failure

**DOI:** 10.1186/s12967-019-1844-0

**Published:** 2019-03-19

**Authors:** Yunyun Wang, Chao Chen, Jinjin Qi, Fengtian Wu, Jun Guan, Zhi Chen, Haihong Zhu

**Affiliations:** State Key Laboratory for Diagnosis and Treatment of Infectious Diseases, Collaborative Innovation Center for Diagnosis and Treatment of Infectious Disease, The First Affiliated Hospital, School of Medicine, Zhejiang University, 79 Qingchun Road, Shangcheng District, Hangzhou, 310003 Zhejiang China

**Keywords:** ACLF, PGE2, EP2, Systemic inflammation

## Abstract

**Background and aims:**

Prostaglandin E receptor 2 (EP2) is an immune modulatory molecule that regulates the balance of immunity. Here we investigated the role of EP2 in immune dysregulation in patients with acute-on-chronic liver failure (ACLF).

**Methods:**

Plasma Progstaglandin E2 (PGE2) levels and EP2 expression on immune cells were determined in blood samples collected from patients with chronic hepatitis B related ACLF(HB-ACLF), patients with chronic hepatitis B (CHB), acute decompensated cirrhosis without ACLF (AD) and healthy controls (HC). Cytokine production, bacterial phagocytosis and reactive oxygen species (ROS) production were detected to explore the role of EP2 in regulating immune cell functions.

**Results:**

The plasma PGE2 levels were increased and EP2 expression on CD8^+^ T cells was decreased in HB-ACLF compared with those in controls. The levels of PGE2 and EP2 were associated with systemic inflammation and disease severity. Small molecular chemicals against EP2 increased both cytokine secretion in PBMCs and ROS production in neutrophils and monocytes, but decreased monocytic phagocytosis. By contrast, an EP2-selective agonist reduced the production of a series of cytokines in PBMCs, but increased G-CSF.

**Conclusion:**

Altered PGE2-EP2 augmented the excessive inflammation of innate and adaptive immune cells in response to LPS or *E. coli* in HB-ACLF. EP2 might be a new potential target for HB-ACLF treatment.

**Electronic supplementary material:**

The online version of this article (10.1186/s12967-019-1844-0) contains supplementary material, which is available to authorized users.

## Background

Hepatitis B virus (HBV) infection, the most common chronic viral infection worldwide, could lead to chronic liver inflammation, cirrhosis and hepatocellular carcinoma [1]. One of the most serious forms is acute-on-chronic liver failure (ACLF), a newly defined syndrome associated with high 28-day mortality [2].

ACLF exhibits remarkable features of exacerbated systemic inflammation that are closely associated with poor outcomes [2]. This systemic response is usually induced by some danger-associated molecular patterns (DAMPs) and pathogen-associated molecular patterns (PAMPs). DAMPs, such as high mobility group box-1 protein (HMGB1), can be released by acute hepatic injury, and PAMPs, such as lipopolysaccharide (LPS) usually translocate from intestine [3]. Both DAMP and PAMP can activate the innate and adaptive immune systems. The excessive inflammatory response to PAMPs or DAMPs can be related to the type of pathogen, site of infection and single-nucleotide polymorphisms (SNPs) in genes encoding molecules involved in the immune response [4]. Recently, IL-33, a DAMP molecule, has been shown to enhance the LPS-induced monocytic inflammatory response in HBV-related ACLF [5]. However, how these DAMPs and PAMPs trigger overwhelming inflammation needs further study.

Inhibitory check points, expressed on immune effector cells, maintain immune balance and prevent an excessive inflammatory response. We have already demonstrated that decreased Tim-3 is associated with the monocytic over response to LPS in decompensated cirrhosis [6]. Prostanoid type E receptor-2 (EP2) and prostanoid type E receptor-4 (EP4) are other immune modulatory receptors, with prostaglandin E2 (PGE2) as their ligand. In chronic viral infection, EP2/4 double-knockout mice demonstrated increased cytokine production of virus-specific cytotoxic T lymphocytes (CTLs) [7]. During influenza A virus infection, increasing PGE2 suppressed the production of type I interferon and apoptosis in macrophages via EP2 and EP4 [8]. Since there is an increase of plasma PGE2 in decompensated cirrhosis, exerting its role as an immunosuppressant on macrophages through EP2 [9]. Nevertheless, EP2 and EP4 can also serve as immune stimulators. Their exact role as immune suppressants or stimulators depends on the micro-environment of the cells, maturation and activation state of the cells, local concentration of PGE2 and whether it is a homeostatic or inflammatory scenario [10]. Thus, the role of EP2 and EP4 in ACLF with an inflammatory storm remains mysterious. Since PGE2 suppress macrophage in acute decompensated cirrhosis through EP2 [9], here we will also focus on EP2 receptors.

This study was designed to investigate whether and how the PGE2-EP2 axis alter innate and adaptive immune function and the response to LPS in patients with hepatitis B- related ACLF (HB-ACLF).

## Methods and materials

### Study subjects

In total, 323 subjects were recruited from outpatients or inpatients of the First Affiliated Hospital of Zhejiang University between October 2016 and December 2017. These subjects were divided into four groups: (i) HB-ACLF: 135 CHB patients with ACLF diagnosed by the criteria of the Asian-Pacific Association for the Study of the Liver (APASL)—i.e., “acute hepatic insult manifesting as jaundice (≥ 5 mg/dL) and coagulopathy, complicated within 4 weeks by ascites and/or encephalopathy in a patient with previously diagnosed or undiagnosed chronic liver disease.” [11]; (ii) acute decompensated cirrhosis (AD): 30 AD patients were characterized by cirrhosis complicated with ascites, hepatic encephalopathy and upper gastrointestinal bleeding. Cirrhosis was defined by previous endoscopy, liver biopsy, radiological evidence, or clinical manifestation of liver decompensation. (iii) chronic hepatitis B (CHB): 128 age-/sex-matched patients with stable chronic hepatitis B were included. Chronic hepatitis B was diagnosed by serum HBsAg positive for more than six months together with histology or imaging or laboratorial or clinical evidence of cirrhosis or liver fibrosis or long-term liver inflammation; (iv) healthy controls (HC): 180 age-/sex-matched healthy subjects were used as controls. Multi-organ failure was diagnosed with two or more extrahepatic organ failures happen according to the chronic liver failure sequential organ failure assessment (SOFA) score. Exclusion criterions were as follows: age younger than 18 years, human immunodeficiency virus infection, pregnancy, immunotherapy, cancer, and a history of autoimmune diseases. Written consent was obtained from each subject or their nominated next of kin if the participants could not provide informed consent. This study was performed according to the principles of the Helsinki Declaration and was approved by the Ethic Committee of the First Affiliated Hospital of Zhejiang University. The baseline characteristics of the patients are shown in (Additional file 1: Table S1). The model for end-stage liver disease (MELD) score and CLIF-consortium organ failure (CLIF-C) score were calculated to assess the severity of the disease.

### Antibodies

CD3-percp-cy5.5 (Catalogue #45-0036-42), CD8-APC (Catalogue #17-0086-42), CD8-FITC (Catalogue #11-0086-42), CD14-APC (Catalogue #17-0149-42), CD56-FITC (Catalogue #4278380), CD16-PerCP-eFluor™710 (Catalogue #46-0168-42), CD11b-PerCP-eFluor™710 (Catalogue #46-0110-80), IFN-γ-PE (Catalogue #12-7319-42), TLR2-FITC (Catalogue #11-9922-41) and Mouse IgG1-PE (Catalogue #12-4714-81) were all bought from eBioscience. HLA-DR-PE (Catalogue #555812), IL-10-APC (Catalogue #554707), CXCR3-APC (Catalogue #550967) and IFN-γ-FITC (Catalogue #561053), Rat IgG2a-APC (Catalogue #554690) and mouse IgG-APC (Catalogue #5065947) were purchased from BD Biosciences. CD16-FITC (Catalogue #302006), HLA-DR-APC (Catalogue #307609), CD3-FITC (Catalogue #317306) and TLR4-APC (Catalogue #312815) were purchased from Biolegend. EP2-PE (Catalogue #10477) and Rabbit IgG-PE (Catalogue D5-1610) were purchased from Cayman Chemical.

### Isolation of peripheral blood mononuclear cells

Whole-blood samples were collected within 24 h after admission. After centrifugation, the plasma was collected and stored immediately at − 80 °C. Peripheral blood mononuclear cells (PBMCs) were isolated using Ficoll-Hypaque density gradient centrifugation (GE healthcare, Pittsburgh, USA).

### Measurement of plasma cytokines and PGE2

The plasma cytokine levels of ACLF patients were measured using a multiplex panel (Bio-Rad, Hercules, CA), according to the manufacturer’s instructions. PGE2 was measured using the Prostaglandin E Metabolites ELISA kit (Cayman Chemical, Michigan, USA). Both measurements were performed according to the manufacturer’s instructions. The detection limits were as follows:IL-1β, 17.3 pg/ml; IL-1rα, 15 pg/ml; IL-2, 16.5 pg/ml; IL-4, 11 pg/ml; IL-5, 27 pg/ml; IL-6, 24.8 pg/ml; IL-7, 11 pg/ml; IL-8, 14.5 pg/ml; IL-9: 21 pg/ml; IL-10: 33.3 pg/ml; IL-12: 15.5 pg/ml; IL-13: 13.5 pg/ml; IL-5; 57.3 pg/ml; IL-17: 24.8 pg/ml; eotaxin, 17 pg/ml; FGF basic, 14 pg/ml; G-CSF, 36.3 pg/ml; GM-CSF, 16 pg/ml; IFN-γ, 15.5 pg/ml; IP-10, 14.5 pg/ml; MCP-1, 18.5 pg/ml; MIP-1α, 8.8 pg/ml; PDGF-bb, 32.5 pg/ml; MIP-1β, 31.5 pg/ml; RANTES, 15.8 pg/ml; TNF-α, 12.5 pg/ml; VEGF, 109.3 pg/ml; PGE2, 2 pg/ml.

### Measurement of cell surface markers

Isolated PBMCs or 100 μl of heparin-treated peripheral whole blood was incubated with antibodies for 15 min at room temperature. After incubation, whole-blood samples were lysed with FACS lysing solution and were washed with phosphate-buffered saline before analysis using an Accuri C6 cytometer (Accuri, BD). The isotype IgG was used as the control.

To explore factors driving EP2 expression, PBMC from healthy controls were incubated for 7 days with a range of IP-10 and MIP-1β and assessed for EP2 expression.

Gating strategies of flow cytometry for immune subsets are introduced in Figure S1 (Additional file 2): CD8^+^T cells (CD3^+^/CD8^+^), CD4^+^T cells (CD3^+^/CD8^−^), NK (CD3^−^/CD56^+^), NKT (CD3^+^/CD56^+^), monocytes (CD14^+^), neutrophils (CD16^+^).

### Intracellular cytokine assays

In vitro, PBMCs (2 × 10^5^/well) were stimulated with lipopolysaccharide (LPS) (100 ng/ml; Sigma-Aldrich, St. Louis, MO) for 72 h in the presence or absence of AH6809 (150 μM; Cayman Chemical) or DMSO (0.1%) in 1640 RPMI medium (Invitrogen, Oslo, Norway) supplemented with 10% fetal bovine serum (FBS, Invitrogen), 100 U/ml of penicillin, and 100 U/ml of streptomycin (Invitrogen) at 37 °C in 5% CO_2_. For the last 6 h, monensin (1.7 μg/ml; Biolegend) was added to the medium to inhibit cytokine secretion, and the cells were re-stimulated with LPS (100 ng/ml). Next, the cells were incubated with fixation/permeabilization solution (BD biosciences) at 4 °C for 20 min. After incubation, the cells were washed, stained with IFN-γ-PE and IL-10-APC for 15 min at room temperature and analyzed by flow cytometry.

### Measurement of supernatant cytokines

In vitro, PBMCs (2 × 10^5^/well) were stimulated with LPS (100 ng/ml; Sigma-Aldrich, St. Louis, MO) for 72 h in the presence or absence of AH6809 (150 μM) or butaprost (50 μM; Sigma) or DMSO in 10% FBS RPMI medium. Next, the supernatants were collected and stored at − 80 °C immediately. Cytokines in the supernatants were measured using a multiplex panel (Bio-Rad, Hercules, CA) according to the manufacturer’s instructions. The limitations of detection have been mentioned above.

### Phagocytosis assay

One hundred microliters of whole blood were incubated with AF488-conjugated *Escherichia coli* (K-12 strain) bio-particles (Molecular probe, Eugene, OR, USA) in 96-well plates and was analyzed by FACS according to previously described protocols [6]. For blocking experiments, the blood was preincubated with AH6809 or DMSO for 1 h at 37 °C in 5% CO_2_.

### Oxidative burst assays

One hundred microliters of whole blood samples were incubated with or without heat-inactivated *E. coli* (8.04*10^7^cfu/ml) or PMA (50 ng/ml) in 96-well plates for 30 min. Next, the cells were assessed for oxidative burst using an ROS assay kit (Genecopoeia, MD, USA) and were analyzed by FACS according to the manufacturer’s protocol. For blocking experiments, blood was pre-incubated with AH6809 or DMSO for 1 h at 37 °C in 5% CO_2_.

### Statistical analysis

The data are shown as the mean ± standard deviation (SD), mean ± standard error of the mean (SEM), median (range) or number (percentage). For comparisons between two independent groups, the Mann–Whitney U test was used. Comparisons between paired groups were performed by the Wilcoxon signed-rank test. Correlations between variables were calculated by the Spearman’s rank correlation test. P < 0.05 at two sides was considered statistically significant. All statistical analyses were performed using GraphPad Prism 6 (GraphPad Software).

## Results

### Hyper inflammatory status in ACLF

We confirmed an elevation of various cytokines levels in HB-ACLF (Additional file 2: Figure S2a). Additionally, spontaneous ROS production in monocytes and neutrophils was significantly enhanced in HB-ACLF than in HC and CHB (Additional file 2: Figure S2bc). No differences were found between CHB and healthy controls. These results were partly consistent with those of a previous report [12].

### An enhanced immune response to Gram-negative bacteria differentiates between ACLF and chronic hepatitis B

**Fig. 1:**
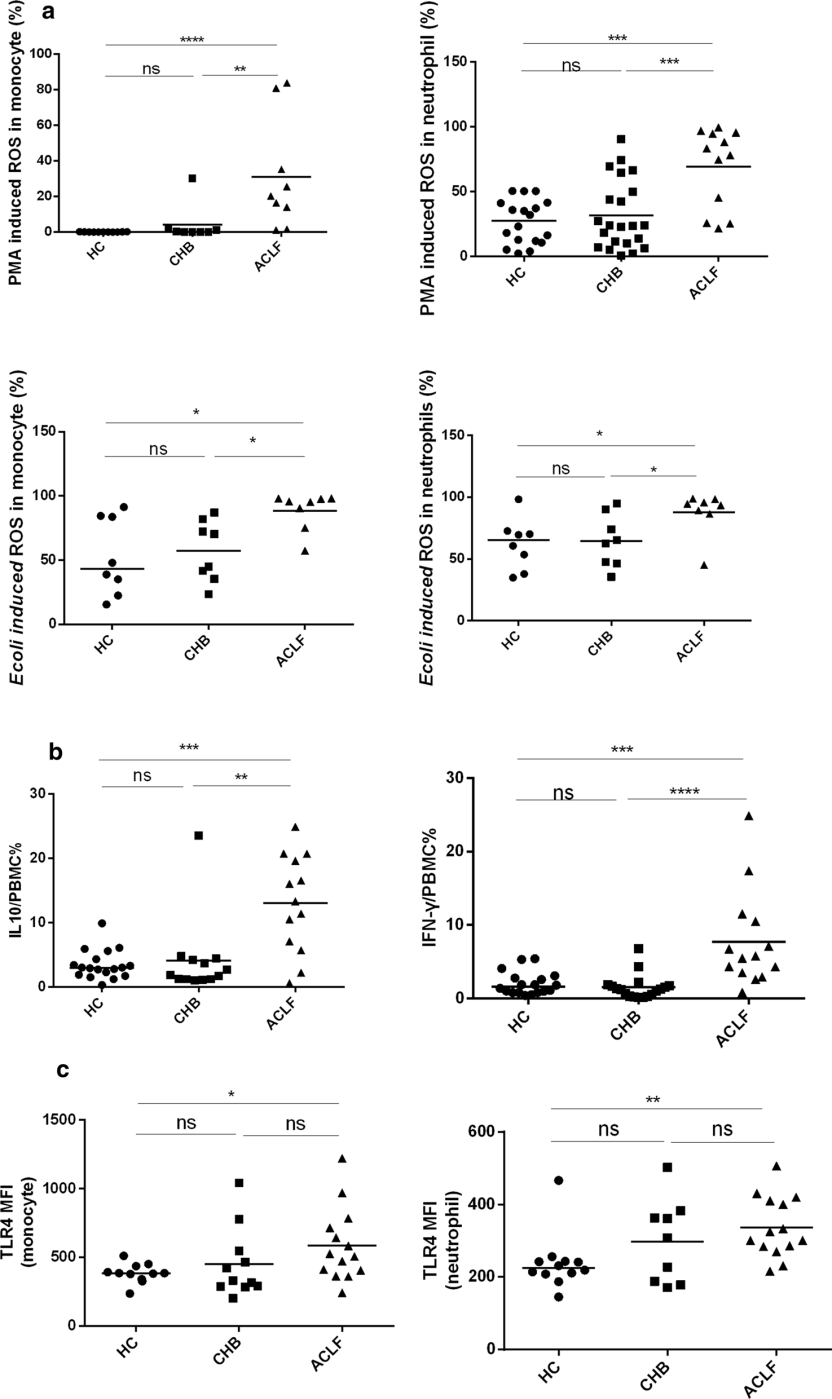
Hypersensitivity of blood immune cells to pro-inflammatory stimulation in patients with ACLF. **a** ROS production in monocytes and neutrophils from HC (n = 18), CHB (n = 22) and ACLF (n = 13) was determined after whole blood was stimulated with PMA (50 ng/ml) or *E. coli*(8.04*10^7^cfu/ml) for 30 min in vitro. **b** Intracellular IL10 and IFN-γ in PBMCs isolated from HC (n = 18), CHB (n = 12) and ACLF (n = 13) were analyzed after PBMCs were stimulated with LPS (100 ng/ml) for 72 h. **c** TLR4 expression on monocytes and neutrophils was determined in HC (n = 12), CHB (n = 9) and ACLF (n = 13). All experiments were performed by flow cytometry. The horizontal line represented the median, and statistical analyses were performed using the Mann–Whitney test. *p < 0.05, **p < 0.01, ***p < 0.001, ****p < 0.0001; ns, not statistically significant

It was reported that endotoxins are significantly elevated in patients with ACLF who can easily develop Gram-negative bacterial infection. These infections are severe and are associated with intense systemic inflammation and poor clinical outcomes [13]. To determine whether endotoxins or Gram-negative bacterial infection is involved in the elevated levels of plasma cytokines, we investigated the immune response of white blood cells to Gram-negative bacteria or LPS. Patients with ACLF showed a remarkable increase in the monocytic and neutrophil ROS production upon PMA and *E. coli* stimulation compared with those of the HC and CHB groups (Fig. 1a). There was a significant increase in the frequency of LPS-stimulated PBMCs producing IL-10 or IFN-γ in ACLF, but not in CHB, compared with healthy controls (Fig. 1b). As expected, in neutrophils and monocytes, TLR4 was significantly upregulated in ACLF only. However, CD16, TLR2 and CD11b were unexpectedly down regulated in patients with ACLF and AD than in patients with CHB and HC (Additional file 2: Figure S3). There was no difference in TLR4 expression on CD8^+^ T cells among the three groups (Additional file 2: Figure S3). Regarding antigen-presenting function, monocytes in ACLF exhibited lower expression of HLA-DR than both HC and CHB (Additional file 2: Figure S3).

To further demonstrate the hypersensitivity of blood immune cells in ACLF, we isolated peripheral monocytes from HC and differentiated them into macrophages by M-CSF. These differentiated macrophages incubated in ACLF plasma exhibited enhanced M1 polarization after LPS stimulation while their ability for M2 polarization was significantly suppressed after IL-4 stimulation compared with macrophages incubated in HC plasma (Additional file 2: Figure S4ab). Such a phenomenon was also seen in THP-1 cell lines (Additional file 2: Figure S4 cd).

### Altered PGE2-EP2 expression in ACLF

**Fig. 2:**
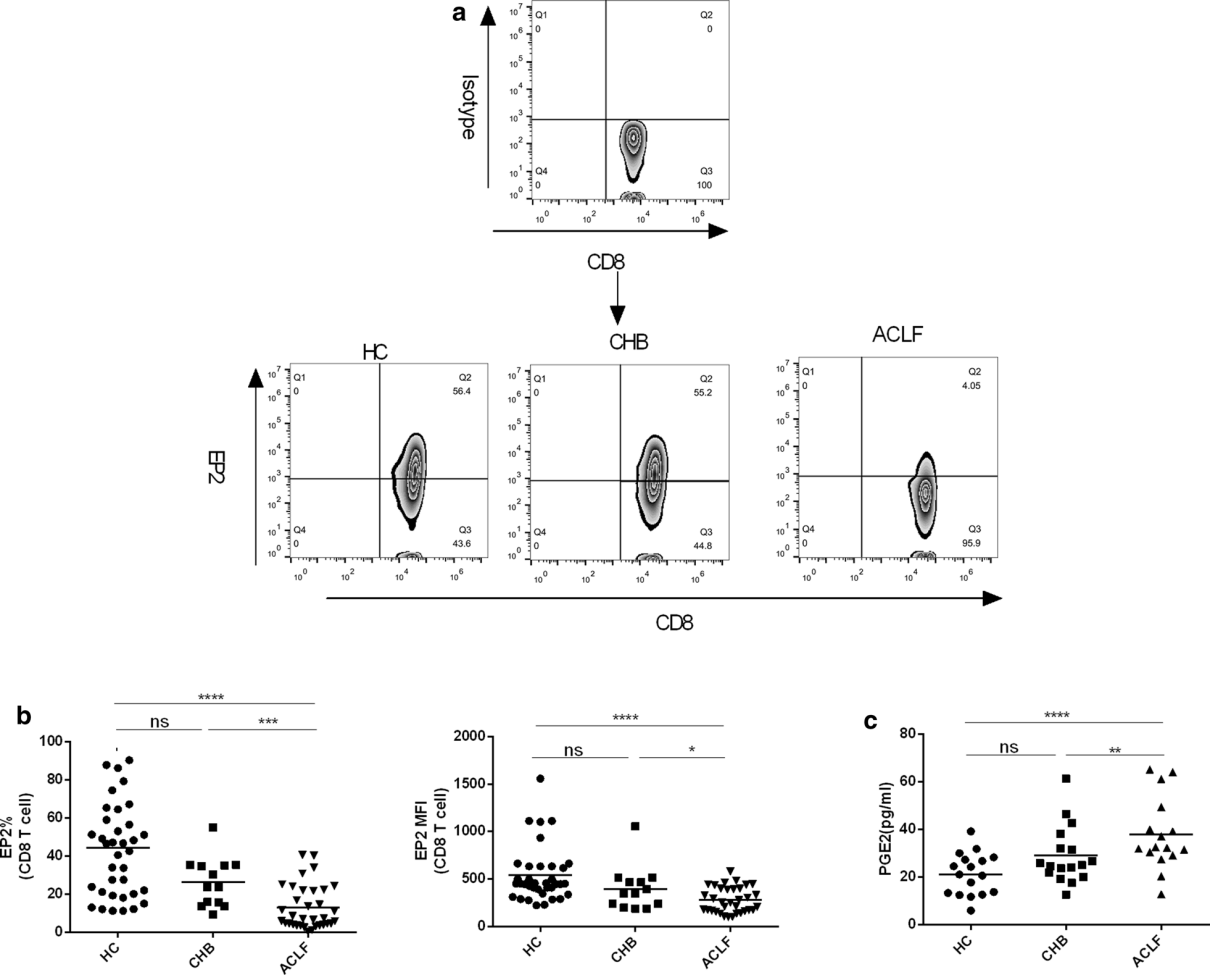
Altered PGE2-EP2 expression in ACLF. **a** Representative plots for EP2 expression on CD8^+^ T cells from four groups. **b** EP2 expression on CD8^+^ T cells in HC (n = 36), CHB (n = 13) and ACLF (n = 34) was determined by flow cytometry. **c** Plasma PGE2 levels were measured in HC (n = 17), CHB (n = 17) and ACLF (n = 16) by ELISA. The horizontal line represented the median, and statistical analyses were performed using the Mann–Whitney test. *p < 0.05, **p < 0.01, ***p < 0.001, ****p < 0.0001; ns, not statistically significant

Next, we assumed that immune inhibitory factors might be involved in the hyper-reaction of blood immune cells to endotoxins or *E. coli*. Thus, we investigated the expression of EP2, an immune modulatory molecule, on the blood innate and adaptive immune cells. As shown in Fig. 2b and Figure S5a, EP2 expression on CD8^+^ T cells were significantly lower in ACLF than in HC and CHB by frequency (p < 0.0001; p = 0.001), MFI (p < 0.0001, p = 0.045) and count (p < 0.0001; p = 0.002). In contrast, no difference of EP2 expression on CD8^+^ T cells was found between CHB and HC. There was a decrease of the count of EP2 positive CD4^+^ T cell and NK cells in ACLF than HC (p = 0.012) and CHB (p = 0.023) respectively (Additional file 2: Figure S5bc). However, EP2 expression on CD4^+^ T cell and NK cells by frequency and MFI showed no significant difference among the three groups (Additional file 2: Figure S5bc). On monocyte, the MFI of EP2 and the count of EP^+^ cells were higher in ACLF than in CHB (p = 0.005; p = 0.007) and HC (p = 0.013; p = 0.038), but no significant differences of the frequency of EP2^+^ monocyte were found between ACLF than HC (p = 0.16) (Additional file 2: Figure S5d). On neutrophils, only MFI of EP2 was increased in ACLF than HC (p = 0.0006) (Additional file 2: Figure S5e). EP2 expression on NKT cells did not differ among these groups by either frequency or MFI or count (Additional file 2: Figure S5f). We also found that plasma PGE2, the ligand of EP2, showed higher levels in ACLF than in HC and CHB (p = 0.0002; p = 0.033, Fig. 2c).

### Altered PGE2-EP2 expression was associated with disease severity in ACLF

**Fig. 3:**
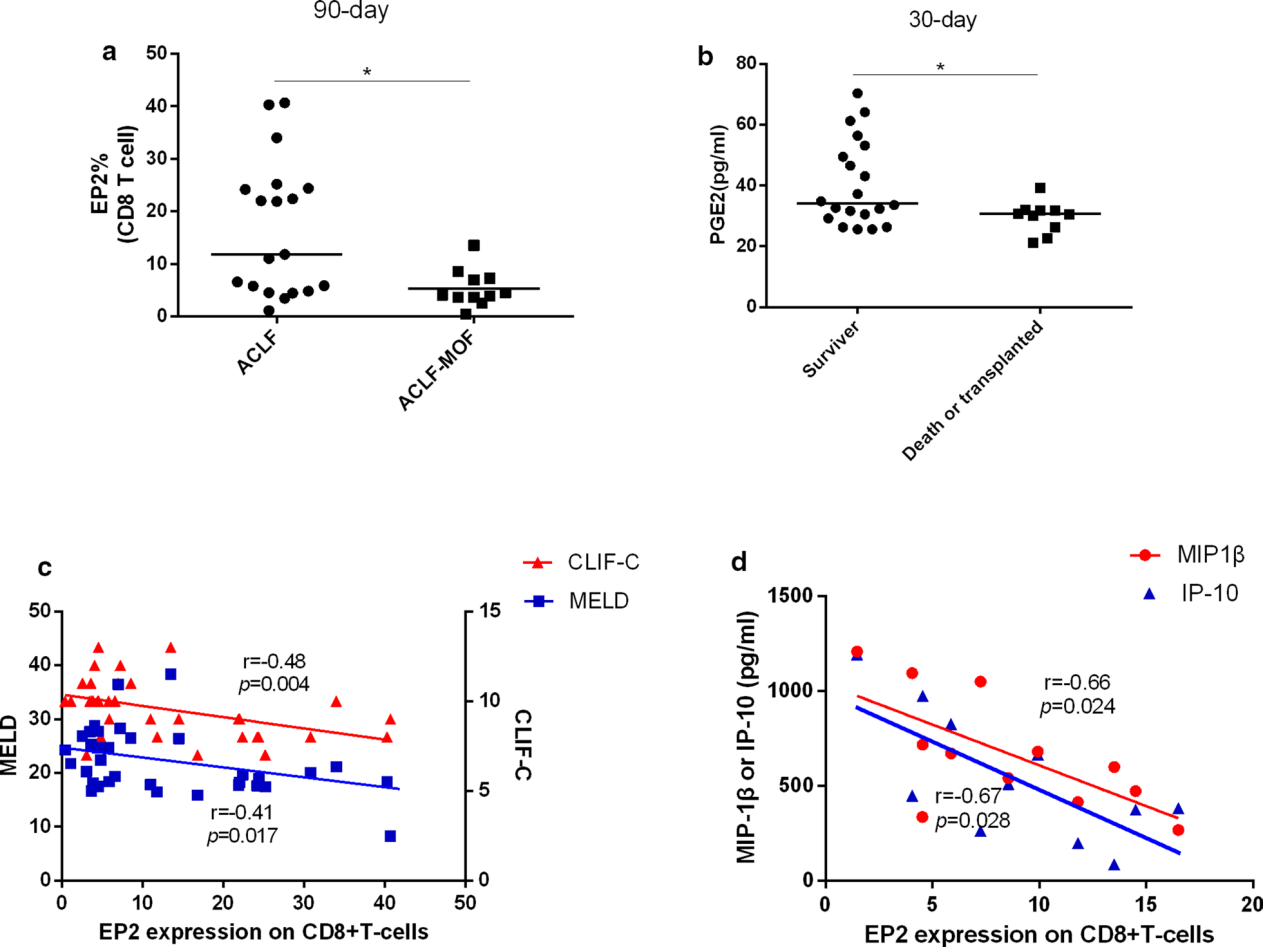
Altered PGE2-EP2 expression is associated with disease severity in ACLF. **a** EP2 expression on CD8^+^ T cells in ACLF (n = 19) and ACLF-MOF (n = 11) was determined by flow cytometry. ACLF-MOF was defined as the development of multi-organ failure within the 90-day follow-up. **b** Plasma PGE2 levels were measured in survivors (n = 20) and the death group (n = 10) during the 30-day follow-up by ELISA. **c** Correlation analysis between EP2 expression and the prognostic score. **d** Correlation analysis between EP2 expression and the plasma cytokine level. The horizontal line represented the median. Statistical analyses were analyzed using the Mann–Whitney test or spearman correlation. *p < 0.05

We further investigated the association of PGE2-EP2 expression with disease severity. Notably, patients with MOF showed decreased frequency of EP2-positive CD8^+^ T cells compared with patients without MOF (Fig. 3a), and ACLF patients who died or received transplantation during the 30-day follow-up had a lower PGE2 level (Fig. 3b). Moreover, a strong negative association was found between the frequency of EP2-positive CD8^+^ T cells and both MELD (r = − 0.41, p = 0.017, Fig. 3c) and CLIF-C scores (r = − 0.48, p = 0.004, Fig. 3c). In addition, the frequency of EP2-positive CD8^+^ T cells was significantly negatively associated with MIP-1β (r = − 0.66, p = 0.024, Fig. 3d) and IP-10 (r =  − 0.67, p = 0.028, Fig. 3d). CXCR3 is the receptor of IP-10. Interestingly, the frequency of CXCR3^+^ CD8^+^ T cells was significantly lower in ACLF than in HC, while CXCR3^+^ monocytes were both higher in CHB and ACLF than in HC, indicating that increased IP-10 in ACLF exerted its function through monocytes but not in CD8^+^ T cells (Additional file 2: Figure S6). Nevertheless, no significant correlation was found between PGE2-EP2 expression and the 90-day mortality (Additional file 2: Figure S7ab). Additionally, the plasma PGE2 level in ACLF showed no correlation with the development of MOF or prognostic score (Figure S7 cd).

All these data indicated an altered PGE2-EP2 axis might be involved in the progression of ACLF.

### MIP-1β together with IP-10 decreased the frequency of EP2^+^CD8^+^T cells

**Fig. 4:**
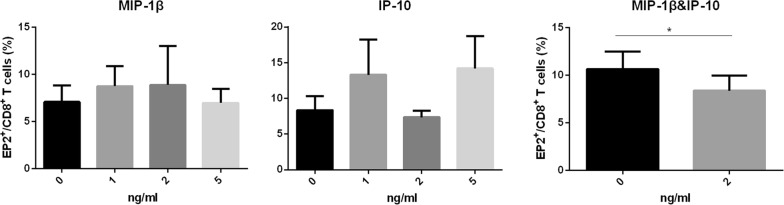
MIP-1β together with IP-10 decreased EP2 expression on CD8^+^T cells. PBMC was isolated from healthy controls (n = 5) and incubated with MIP-1β (1 ng/ml, 2 ng/ml, 5 ng/ml), IP-10 (1 ng/ml, 2 ng/ml, 5 ng/ml) or a combination of MIP-1β and IP-10 (2 ng/ml) for 7 days. The frequency of EP2^+^ CD8^+^ T cells was determined by flow cytometry. Statistical analyses were analyzed using the paired t test. *p < 0.05

Then, we investigated whether MIP-1β or IP-10 decreased the frequency of EP2^+^CD8^+^T cells. After incubated with the combination of MIP-1β and IP-10 for 7 days, PBMC isolated from HC showed decreased frequency of EP2^+^CD8^+^T cells (Fig. 4). However, there was no change in the frequency of EP2^+^CD8^+^T cells when incubated with MIP-1β or IP-10 alone (Fig. 4).

### Characterization of EP2-positive immune cells

**Fig. 5:**
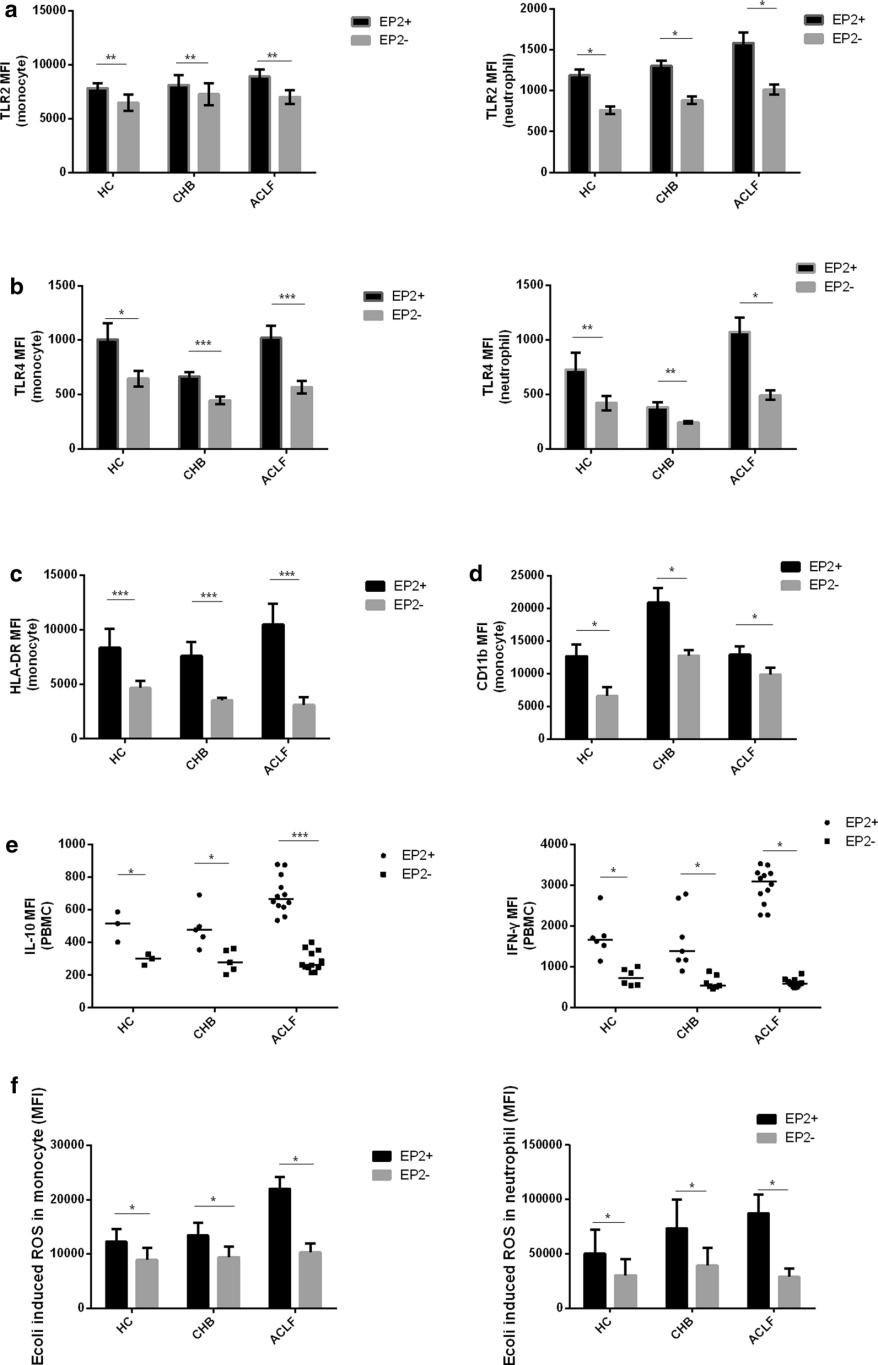
Characterization of EP2-positive immune cells. **a**–**d** Peripheral whole blood (at least 5 subjects per group) was stained with anti-CD14, anti-CD16, anti-EP2, anti-TLR2, anti-HLA-DR, anti-CD11b or anti-TLR4. The percentages of different phenotypes of EP2^+^ or EP2^−^ monocytes and neutrophils were determined by flow cytometry. **e** PBMCs isolated from at least 3 subjects per group were stimulated with LPS (100 ng/ml) for 72 h. Cytokine production was measured by flow cytometry. **f** Peripheral whole blood from 7 subjects per group was incubated with *E. coli* for 30 min. ROS production in neutrophils and monocytes was assessed by flow cytometry. The data are expressed as the mean ± SEM of individual subjects or plots of individual data. The error bar represented SEM and the horizontal line represented the median. Statistical analyses were analyzed using the Wilcoxon signed-rank test. *p < 0.05, **p < 0.01, ***p < 0.001; ns, not statistically significant

To determine the function of EP2-positive immune cells, we compared the phenotypes of EP2-positive cells with those of EP2-negative immune cells. Among all three groups, EP2-positive neutrophils and monocytes showed higher TLR2 and TLR4 expression than EP2-negative ones (Fig. 5a, b), indicating a higher response of EP2-positive cells to TLR4 and TLR2 ligands, such as LPS. There was also a marked increase in HLA-DR and CD11b expression on EP2^+^ monocytes compared with that on EP2- (Fig. 5c, d). Additionally, IFN-γ and IL-10 production in response to LPS was stronger in EP2^+^ PBMCs than in EP2- (Fig. 5e). After whole blood was stimulated with *E. coli* in vitro, ROS production was enhanced in EP2^+^ monocytes and neutrophils than in EP2^−^ ones (Fig. 5f). Intriguingly, the difference in these phenotypes between EP2^+^ immune cells and EP2^−^ immune cells seemed always larger in the ACLF group than in the HC and CHB groups, suggesting an immune imbalance in ACLF. These phenomena suggested an immune-activation role of EP2-positive immune cells.

### Blockade of EP2 enhances the immune response in ACLF

**Fig. 6:**
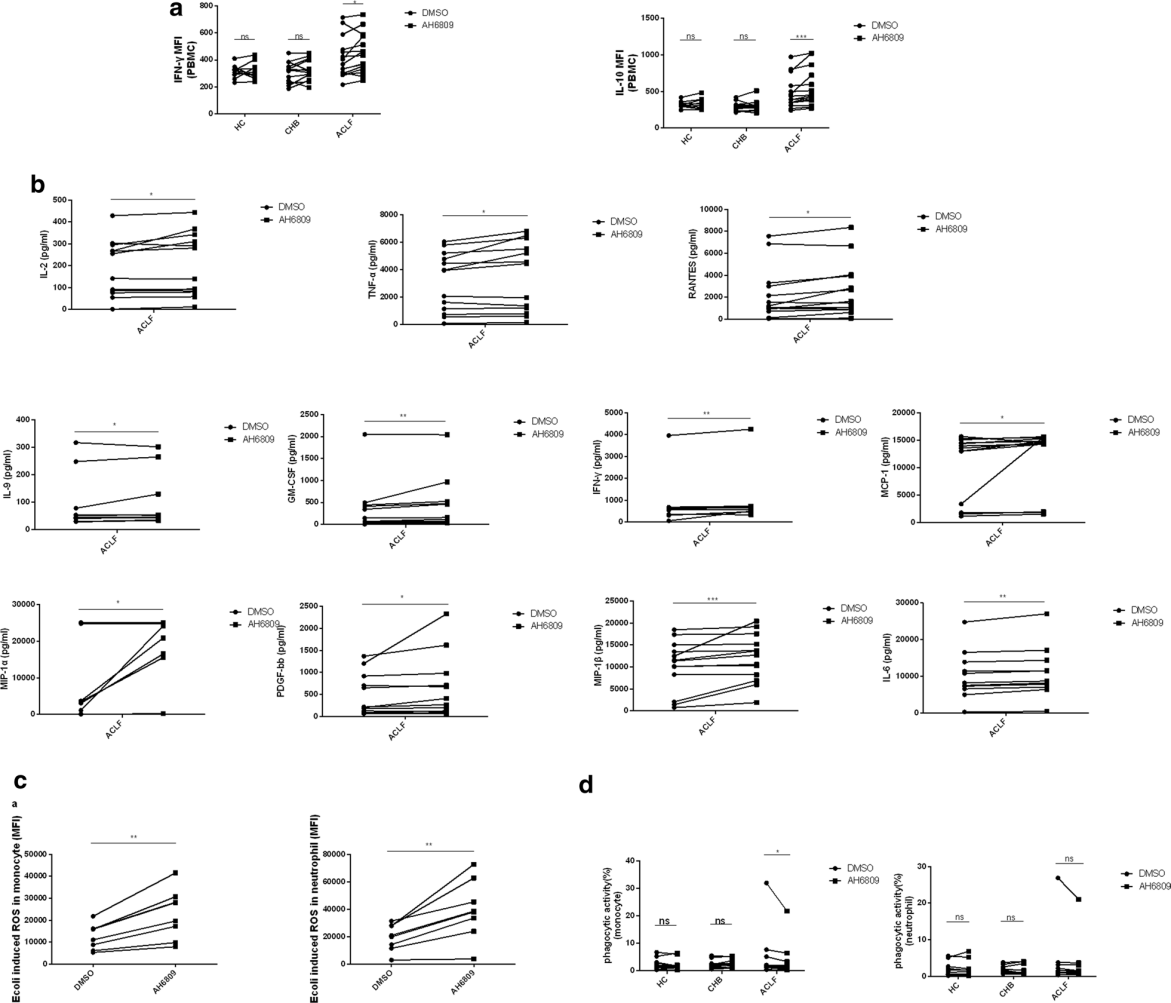
Blockade of EP2 enhances the immune response in ACLF. Isolated PBMCs from at least 9 subjects per group were stimulated with LPS in the presence of AH6809 (150 μM) or DMSO for 72 h in vitro. The impact of EP2 blockade on cytokine production was determined by flow cytometry (intracellular staining) and multi-plex human cytokine assay (supernatants). **a** Percentage of cytokines producing PBMCs. **b** Concentration of cytokines in the supernatants. **c** One hundred microliters of peripheral blood from 8 subjects per group was stimulated with *E. coli* in the presence of AH6809 (150 μM) or DMSO for 30 min in vitro. The effect of EP2 blockade on ROS production of monocytes and neutrophils was determined by flow cytometry. **d** One hundred microliters of peripheral blood from HC (n = 10), CHB (n = 12) and ACLF (n = 10) was incubated with *AF488*-*E. coli* in the presence of AH6809 (150 μM) or DMSO for 30 min in vitro. The effect of EP2 blockade on the phagocytosis of monocytes and neutrophils was determined by flow cytometry. Statistical analyses were performed using the Wilcoxon signed-rank test. *p < 0.05, **p < 0.01, ***p < 0.001. ns, not statistically significant

To explore the exact role of EP2 in immune cells in ACLF patients, we blocked EP2 and observed the change in cytokines, ROS production and phagocytosis. Blockade of EP2 on PBMCs from ACLF but not from CHB or HC could increase the secretion of IFN-γ and IL-10 (Fig. 6a). Moreover, this blockade also increased the secretion of many other cytokines, such as IL-6, TNF-α, and MCP-1 in ACLF (Fig. 6b). Additionally, ROS production in monocytes and neutrophils from ACLF patients was markedly enhanced after EP2 blockade in vitro (Fig. 6c). However, such blockade decreased monocytic phagocytosis in the ACLF group but not in the HC or CHB group (Fig. 6d). No significant change was seen in the phagocytosis of neutrophils (Fig. 6d).

### Activation of EP2 exhibits anti-inflammatory responses and increase G-CSF secretion

**Fig. 7:**
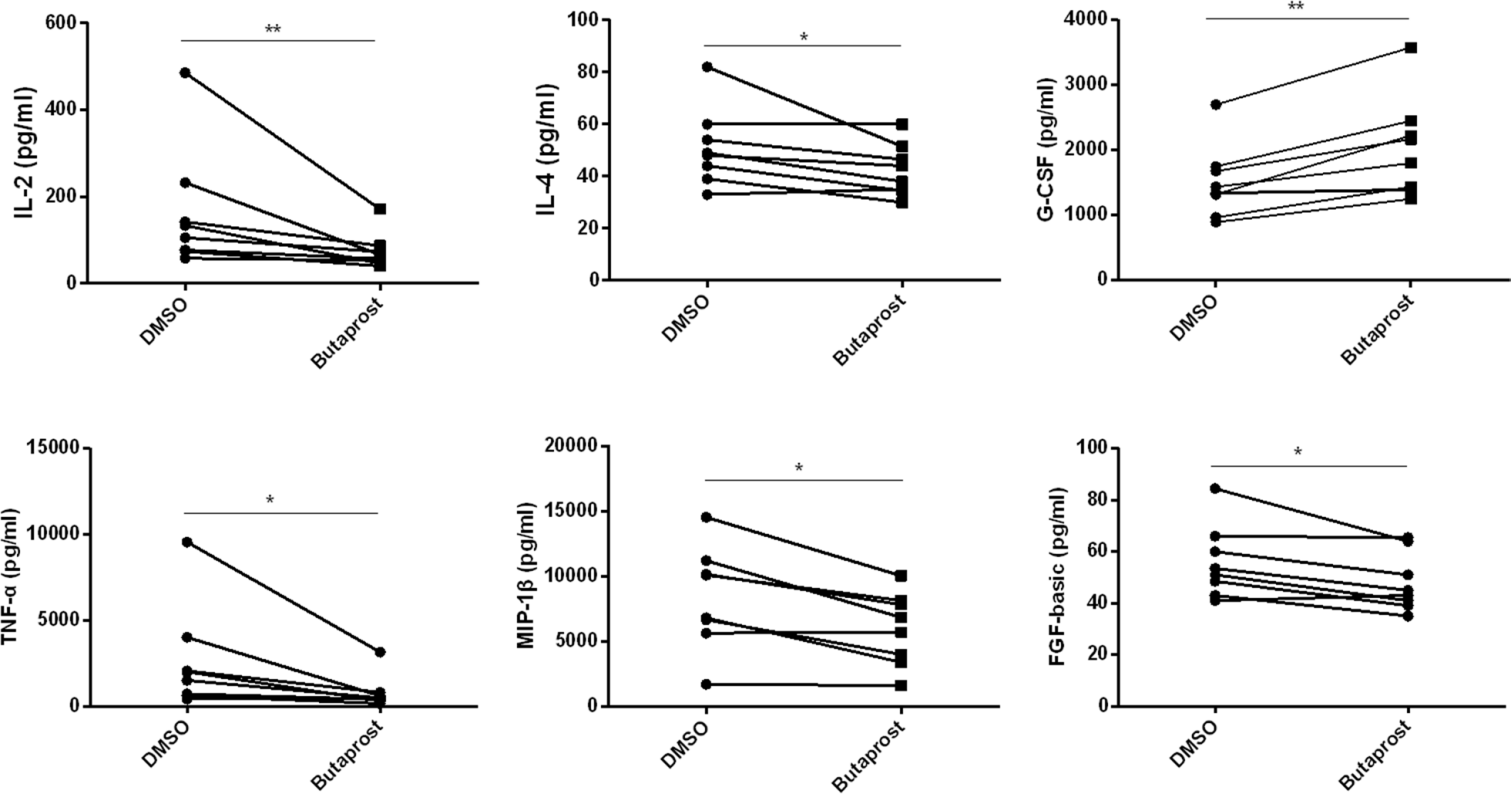
The EP2 agonist inhibits PBMC cytokine production in ACLF. Isolated PBMCs from 8 subjects per group were stimulated with LPS in the presence of butaprost (50 μM) or DMSO for 72 h in vitro. The effect of butaprost on cytokine production was determined by the multiplex human cytokine assay (supernatants). Statistical analyses were performed using the Wilcoxon signed-rank test. *p < 0.05, **p < 0.01

To explore potential drugs for the treatment of ACLF, we employed butaprost, an EP2-selective agonist, to PBMCs isolated from patients with ACLF. There was a significant decrease in IL-2, IL-4, TNF-α, MIP-1β and FGF basic in the supernatants after EP2 was agonized under stimulation of LPS, indicating an anti-inflammatory role of butaprost in ACLF (Fig. 7). In the meantime, there was also a marked increase of G-CSF (Fig. 7), a cytokine that have been proved to promote liver regeneration [14].

## Discussion

Our study found that ACLF patients showed a hyper-inflammatory status, altered phenotypes presenting increased monocytic and neutrophil TLR4 expression and ROS production, and down-regulated monocytic HLA-DR and TLR2. This hyper-inflammatory status was consistent with that in previous reports [15] and was believed to lead to the development of multi-organ failure and high short-term mortality [4]. However, the underlying mechanism remains unclear. The present study found that blood immune cells were hypersensitive to LPS and *E. coli*, very likely to be the cause of the hyper-inflammatory status. Furthermore, an altered PGE2-EP2 axis was found to associate with such immune abnormality and disease severity. The blockade of EP2 resulted in an increase in LPS-stimulated cytokine production in PBMCs, and *E. coli* induced ROS production in both monocytes and neutrophils with impairment in phagocytosis in monocytes. Thus, EP2 might play a key role in regulating the immune system during the pathogenic process of ACLF.

The systemic inflammation in ACLF was well recognized. However, regarding the immune activity of immune cells, divergence occurs. Some studies have shown that patients with ACLF or acute decompensated cirrhosis or acute alcohol hepatitis had increased numbers of immune-regulatory immune cells that suppressed the immune response to LPS compared with HC and chronic liver disease [9, 15, 16]. By contrast, others have found that monocytes from ACLF or decompensated cirrhosis were hypersensitive to LPS stimulation [5, 6]. Interestingly, studies supporting the immunosuppressive state in ACLF or decompensated cirrhosis were all conducted in western countries, and most patients had alcohol-induced liver diseases. However, two studies performed in China showed that hepatitis B infection was the main pre-existing chronic liver disease before the development of ACLF. Another reason might be that the stages of patients recruited were different across the studies. The early stage of sepsis exhibits pro-inflammatory features, and the late stage shows immunosuppressive characteristics. ACLF might also have different immune states in different stages. Among patients with ACLF in China, the major lethal complication was hepatic encephalopathy, but not infection-related disease, indicating the pathogenesis was more closely related to a systemic inflammatory response [17]. Moreover, TLR4 was up-regulated in monocytes and T cells in HBV-related ACLF in China [18, 19], supporting the possibility of the over-response of these immune cells to LPS or *E. coli*. Therefore, our findings that immune cells in HB-ACLF were hypersensitive to LPS or *E. coli* were reliable. This theory also supported the recommendation that a prophylaxis of antibiotics might be necessary because bacterial infection could over-activate the immune response. However, the underlying mechanism was unclear. Thus, our study focused on the potential role of EP2 in the pathogenesis of the systemic inflammatory in ACLF.

We discovered the association of EP2 with disease severity. A lower frequency of EP2^+^ CD8^+^ T cells was found in patients with multi-organ failure and those who died or received liver transplantations within a 30-day follow-up. This frequency was also significantly correlated with the prognostic score. Therefore, we suggested a novel biomarker to predict the disease outcome and immune conditions. A further prospective study needs to be performed on a larger cohort to assess this ability.

We also found patients with ACLF in the death or liver transplantation group had lower plasma PGE2 levels than those in the survival group. During acute liver inflammation or ACLF, many hepatocytes underwent death, releasing DAMP to trigger further inflammation and inhibitory DAMP such as PGE2 to resolve the inflammation [9, 20–22]. The secretion of PGE2 is increased under cell death-inducing conditions through the induction of the gene of cyclooxygenase 2 (COX2) that was a potential target for drug development to increase the production of PGE2 [22]. Thus, patients with ACLF were prone to death if they had insufficient PGE2 to inhibit excessive inflammation.

LPS was well recognized to stimulate innate immunity. However, when LPS stimulates PBMC in vitro, approximately 60% of the LPS-challenged IL-10 and IFN- γ responses were lymphocyte-specific **[**16**]**. Blockade of PD-1 and Tim-3 significantly affected cytokine secretion in CD8 + T cells **[**16**]**. Thus, we used LPS to stimulate PBMCs that consisted of CD8^+^ T cells to test the role of down-regulated EP2 on cytokine secretion.

Expectedly, IL-6 was elevated after EP2 blockage under LPS stimulation. It was reported IL-6 more closely correlated with disease severity in ACLF [12]. In fact, IL-6 is a potent inducer of the acute phase response [2]. These data might indicate the important role of EP2 in the early pathogenesis of ACLF. There was a significant elevation of IP-10 in the plasma of ACLF that was negatively correlated with EP2 expression on CD8^+^ T cells. Nevertheless, its receptor, CXCR3, was up-regulated in monocytes, not CD8^+^ T cells, suggesting that a high plasma concentration of IP-10 strongly induces monocyte signaling. Further blockade of EP2 on PBMCs did not lead to an increase in IP-10 secretion, excluding the possibility that EP2 regulated IP-10 secretion. Except for IP-10, EP2 expression on CD8^+^ T cells was also negatively correlated with MIP**-**1β, and the blockade of EP2 augmented the secretion of MIP-1β, indicating down-regulated EP2 resulted in the elevation of the MIP-1β level in the plasma of patients with ACLF.

This study also found that both spontaneous and stimulated ROS of peripheral monocytes and neutrophils are increased in ACLF patients. The production of ROS is a key factor for activated Kupffer cells to recruit activated neutrophils and monocytes to liver in liver injury. In addition to recruiting immune cells, ROS itself is a toxic medium through which inflammatory cells kill targets, such as bacteria, as well as liver cells and cells from other organs. During the inflammatory response, ROS-induced cytocidal mechanisms include the promotion of mitochondrial dysfunction. Through intracellular oxidative stress in cells, cell damage increases the release of cellular contents, thereby further expanding inflammatory damage [23]. ROS also promotes the secretion of cytokines, which, in turn, cause an increase in ROS production, resulting in a vicious circle and promoting the pathogenesis of liver diseases [24]. Moreover, resting ROS ≥ 12% predicted the 90-day mortality in patients with cirrhosis with high sensitivity and specificity [25]. Together, these data indicated the crucial role of ROS in the pathogenesis in ACLF. Our study found that, in ACLF, ROS production increased significantly after EP2 blockade, suggesting EP2 plays an important role in the regulation of ROS production.

Currently, no specific drug is available to cure this disease. Corticosteroids have been used to relieve inflammation, but they add the risk of infection and their ability to improve mortality remains controversial. Strategies specifically targeting one inhibitory molecule on immune cells might be beneficial to control the overwhelming inflammation with fewer side effects. Having identified PGE2-EP2 as the inhibitory signals in the immune cells and with EP2-selective agonists available [26, 27], we assessed its candidacy as a promising future immunotherapeutic target.

We noticed a particular cytokine-G-CSF was increased after butaprost treatment. G-CSF is a cytokine secreted by epithelial cells and various immune cells, inducing the proliferation and differentiation of granulocytes in the bone marrow. After liver injury, this cytokine is released by bone marrow-derived circulating pluripotent cells to enhance liver regeneration [14]. In a parallel trial for ACLF patients, G-CSF is also found to reduce prognostic score and incidence of serious complications, and increase survival rare [28]. This benefit is thought to be mediated by the migration of CD34^+^ hematopoietic progenitor cells to the liver. Furthermore, after treatment with G-CSF, the production of IFN-γ is decreased in CD8^+^ T cells [14, 29, 30]. In ALF, G-CSF is found to improve phagocytosis and the bactericidal function of neutrophil damage [31, 32]. Our study found that butaprost significantly increased G-CSF secreted by PBMCs in ACLF patients, suggesting butaprost might also have a good therapeutic effect on patients with ACLF.

## Conclusion

Overall, our study discovered that dysfunctional circulating immune cells were present in patients with ACLF. This dysfunction was manifested by an over-reaction to LPS and *E. coli*. The down-regulated EP2 signaling may increase the responsiveness of immune cells to stimuli. Our study provides a possible target for the treatment of excessive systemic inflammation in ACLF.

## Highlights

- EP2 expression on CD8^+^ T cells was decreased in HB-ACLF compared with those in controls.
- The levels of PGE2 and EP2 were associated with systemic inflammation and disease severity.
- Small molecular chemicals against EP2 increased both cytokine secretion in PBMCs and ROS production in neutrophils and monocytes.
- EP2-selective agonist reduced the production of a series of cytokines in PBMCs, but increased G-CSF. EP2 might be a new potential target for HB-ACLF treatment.

## Additional files

**Additional file 1: Table S1.** Baseline characteristics of included subjects.

**Additional file 2: Figure S1.** Gating strategies for various immune subsets. (a) In the density plots of forward and lateral angular scatter, the human whole-blood gates were Lym (lymphocyte), Mo (monocyte) and Ne (neutrophil). (b) CD8^+^ T cells were located at the Lym gate and were double positive for CD3 and CD8, whereas CD3^+^CD8^−^ cells were considered as CD4^+^ T cells in this experiment. (c) NK and NKT cells were also located in the Lym gate and were further confirmed by CD56 and CD3 markers. (d) Monocytes were present in the Mo-gate and were positive for CD14. (e) Neutrophils were present in the Ne-gate and were positive for CD16. **Figure S2.** Hyper inflammatory status in ACLF. (a) Plasma cytokines of ACLF (n = 24), CHB (n = 25) and HC (n = 24) were determined using a multi-plex cytokine assay. (b) Representative plots for spontaneous ROS production in monocytes and neutrophils from three groups. (c) Spontaneous ROS production in monocytes and neutrophils from HC (n = 19), CHB (n = 19) and ACLF (n = 15) was assessed. The error bar represented the SEM and the horizontal line represented the median. Statistical analyses were performed using the Mann–Whitney test. *p < 0.05, **p < 0.01, ***p < 0.001, ****p < 0.0001; ns, not statistically significant. **Figure S3.** Phenotype characterization of blood immune cells. Peripheral whole blood (at least 5 subjects each group) was stained with anti-CD14, anti-CD16, anti-CD3, anti-CD8, anti-TLR2, anti-HLA-DR, anti-CD11b or anti-TLR4. The percentages of different phenotypes of blood immune cells were determined by flow cytometry. The horizontal line represented the median. Statistical analyses were analyzed using the Mann–Whitney test. Four groups were compared with each other. *p < 0.05, **p < 0.01, ***p < 0.001, ****p < 0.0001. **Figure S4.** Plasma from ACLF promotes macrophage M1 transformation. (a-b) Peripheral monocytes from HC were differentiated into macrophage in vitro by the addition of M-CSF for 7 days. Differentiated macrophages from HC were incubated in 20% ACLF plasma or HC plasma for 24 h in the presence of (a) LPS (100 ng/ml) or (b) IL-4 (15 ng/ml). M1 phenotypes were assessed by the expression of CD80, CD86 and HLA-DR, and M2 phenotypes were assessed by the expression of CD206 and CD163. (c-d) PMA-differentiated THP-1 was incubated in 20% ACLF plasma or HC plasma for 24 h in the presence of (c) LPS (100 ng/ml) or (d) IL-4 (15 ng/ml). M1 phenotypes were assessed by the expression of CD86, and M2 phenotypes were assessed by PD-1. All experiments were analyzed by flow cytometry. The horizontal line represented the mean. Statistical analyses were performed using the Mann–Whitney test. *p < 0.05, **p < 0.01, ***p < 0.001. ns, not statistically significant. **Figure S5.** EP2 expression on other immune cells. The horizontal line represented the median, and statistical analyses were performed using the Mann–Whitney test. *p < 0.05, **p < 0.01. ns, not statistically significant. **Figure S6.** Frequency of CXCR3^+^ CD8^+^ T cells and CXCR3^+^ monocytes. The horizontal line represented the median. Statistical analyses were analyzed using the Mann–Whitney test. *p < 0.05, **p < 0.01. ns, not statistically significant. **Figure S7.** Association of PGE2-EP2 expression with disease severity in ACLF. (a) EP2 expression on CD8^+^ T cells in survivors or death or transplantation group during the 90-day follow-up. (b) Plasma PGE2 levels were measured in survivors or death or transplantation group during the 90-day follow-up. (c) Plasma PGE2 levels were measured in ACLF or ACLF-MOF during the 90-day follow-up. (d) Correlation analysis between the plasma PGE2 levels and prognostic score. The horizontal line represented the median. Statistical analyses were analyzed using the Mann–Whitney test or spearman correlation. ns, not statistically significant.

## Abbreviations

HB-ACLF: HBV-related acute-on-chronic liver failure
EP2: Prostaglandin E receptor 2
PGE2: Progstaglandin E2
CHB: chronic hepatitis B
HC: healthy controls
ROS: reactive oxygen species
HBV: Hepatitis B virus
DAMPs: danger-associated molecular patterns
PAMPs: pathogen-associated molecular patterns
HMGB1: high mobility group box-1 protein
LPS: lipopolysaccharide
CTLs: cytotoxic T lymphocytes
APASL: Asian-Pacific Association for the Study of the Liver
MELD: model for end-stage liver disease
CLIF-C: CLIF-consortium organ failure
PBMC: Peripheral blood mononuclear cells
AD: acute decompensated cirrhosis
