# Supplementary material for: Altered PGE2-EP2 is associated with an excessive immune response in HBV-related acute-on-chronic liver failure
Source: J Transl Med. 2019 Mar 19;17:93. doi: 10.1186/s12967-019-1844-0 (PMC6425563; doi:10.1186/s12967-019-1844-0)
Supplement: Supplementary file 1 — Additional file 1: Table S1. Baseline characteristics of included subjects. [file 12967_2019_1844_MOESM1_ESM.docx]

**Table S1.** **Baseline characteristics of included subjects.**

|  | ACLF (n=135) | AD (n=30) | CHB (n=128) | HC (n=180) |
| --- | --- | --- | --- | --- |
| Age (years), Mean (±SD) | 46.1 ± 12.8 | 50.2±11.5 | 45.7 ± 8.5 | 40 ± 11.5 |
| Male (%) | 122 (89.1) | 23 (76.7) | 109 (85.2) | 165 (91.7) |
| **Etiology** |  |  |  |  |
| HBV (%) | 134 (100) | 30(100) | 128 (100) | - |
| **Clinical feature** |  |  |  |  |
| Ascites (%) | 101 (73.7) | 28(93.3) | 0 (0) | - |
| Cirrhosis (%) | 62 (45.3) | 30(100) | 0 (0) | - |
| UGIB (%) | 14 (10.2) | 5(16.7) | 0 (0) | - |
| HE (%) | 11 (8.0) | 2 (6.7) | 0 (0) | - |
| **Laboratory data** |  |  |  |  |
| WBC (×10^9^/L), Median (IQR) | 7.0 (5.1-9.1) | 5.2 (4.2-7.5) | 6.8 (4.6 -8.2) | 6.2 (4.5-6.8) |
| ALT (IU/L), Median (IQR) | 208 (90-601) | 45 (35-75) | 34 (30.0 -65.5) | n.a. |
| Albumin (g/L), Mean (±SD) | 31.1 (28.6-34.0) | 28.9 ± 4.6 | 36.5 ± 4.2 | n.a. |
| Bilirubin (mg/dL), Median (IQR) | 18.6 (12.7-23.8) | 10.2 (5.2-15.5) | 1.29 (1.10-1.55) | n.a. |
| INR，Median (IQR) | 2.0 (1.7-2.6) | 1.35 (1.15-1.55) | 1.15 (1.02- 1.25) | n.a. |
| Creatinine (mg/dL), Median (IQR) | 0.7 (0.7-0.9) | 0.7 (0.6-0.8) | 0.7 (0.6-0.8) | n.a. |
| **Scores** |  |  |  |  |
| CTP, Mean (±SD) | 11.0 ± 1.3 | 10 ± 1.5 | 5.5 ± 1.2 | n.a. |
| MELD, Mean (±SD) | 23.1 ± 6.3 | 10.6 ± 2.9 | 5.1 ± 1.3 | n.a. |
| CLIF-C, Mean (±SD) | 9.8 ± 1.9 | n.a | n.a | n.a. |

ACLF acute-on-chronic liver failure, AD decompensated cirrhosis, CHB chronic hepatitis B, HC healthy controls, UGIB upper gastrointestinal bleeding, HRS hepatorenal syndrome, HE hepatic encephalopathy, SBP spontaneous bacterial peritonitis, WBC white blood cell count., INR international normalized ratio, CTP Child—Turcotte-Pugh, MELD Model for End-stage Liver Disease, CLIF-C chronic liver failure-consortium organ failure.
