# Supplementary material for: Altered PGE2-EP2 is associated with an excessive immune response in HBV-related acute-on-chronic liver failure
Source: J Transl Med. 2019 Mar 19;17:93. doi: 10.1186/s12967-019-1844-0 (PMC6425563; doi:10.1186/s12967-019-1844-0)
Supplement: Supplementary file 2 — Additional file 2: Figure S1. Gating strategies for various immune subsets. (a) In the density plots of forward and lateral angular scatter, the human whole-blood gates were Lym (lymphocyte), Mo (monocyte) and Ne (neutrophil). (b) CD8+ T cells were located at the Lym gate and were double positive for CD3 and CD8, whereas CD3+CD8− cells were considered as CD4+ T cells in this experiment. (c) NK and NKT cells were also located in the Lym gate and were further confirmed by CD56 and CD3 markers. (d) Monocytes were present in the Mo-gate and were positive for CD14. (e) Neutrophils were present in the Ne-gate and were positive for CD16. Figure S2. Hyper inflammatory status in ACLF. (a) Plasma cytokines of ACLF (n = 24), CHB (n = 25) and HC (n = 24) were determined using a multi-plex cytokine assay. (b) Representative plots for spontaneous ROS production in monocytes and neutrophils from three groups. (c) Spontaneous ROS production in monocytes and neutrophils from HC (n = 19), CHB (n = 19) and ACLF (n = 15) was assessed. The error bar represented the SEM and the horizontal line represented the median. Statistical analyses were performed using the Mann–Whitney test. *p < 0.05, **p < 0.01, ***p < 0.001, ****p < 0.0001; ns, not statistically significant. Figure S3. Phenotype characterization of blood immune cells. Peripheral whole blood (at least 5 subjects each group) was stained with anti-CD14, anti-CD16, anti-CD3, anti-CD8, anti-TLR2, anti-HLA-DR, anti-CD11b or anti-TLR4. The percentages of different phenotypes of blood immune cells were determined by flow cytometry. The horizontal line represented the median. Statistical analyses were analyzed using the Mann–Whitney test. Four groups were compared with each other. *p < 0.05, **p < 0.01, ***p < 0.001, ****p < 0.0001. Figure S4. Plasma from ACLF promotes macrophage M1 transformation. (a-b) Peripheral monocytes from HC were differentiated into macrophage in vitro by the addition of M-CSF for 7 days. Differentiated macrophag [file 12967_2019_1844_MOESM2_ESM.docx]

 **Figure S1 Gating strategies for various immune subsets.** (a) In the density plots of forward and lateral angular scatter, the human whole-blood gates were Lym (lymphocyte), Mo (monocyte) and Ne (neutrophil). (b) CD8^+^ T cells were located at the Lym gate and were double positive for CD3 and CD8, whereas CD3^+^CD8^-^ cells were considered as CD4^+^ T cells in this experiment. (c) NK and NKT cells were also located in the Lym gate and were further confirmed by CD56 and CD3 markers. (d) Monocytes were present in the Mo-gate and were positive for CD14. (e) Neutrophils were present in the Ne-gate and were positive for CD16.



 **Figure S2 Hyper inflammatory status in ACLF.** (a) Plasma cytokines of ACLF (n=24), CHB (n=25) and HC (n=24) were determined using a multi-plex cytokine assay. (b) Representative plots for spontaneous ROS production in monocytes and neutrophils from three groups. (c) Spontaneous ROS production in monocytes and neutrophils from HC (n=19), CHB (n=19) and ACLF (n=15) was assessed.

The error bar represented the SEM and the horizontal line represented the median. Statistical analyses were performed using the Mann–Whitney test. *p<0.05, **p<0.01, ***p<0.001, ****p<0.0001; ns, not statistically significant.



 **Figure S3 Phenotype characterization of blood immune cells.**

Peripheral whole blood (at least 5 subjects each group) was stained with anti-CD14, anti-CD16, anti-CD3, anti-CD8, anti-TLR2, anti-HLA-DR, anti-CD11b or anti-TLR4. The percentages of different phenotypes of blood immune cells were determined by flow cytometry. The horizontal line represented the median. Statistical analyses were analyzed using the Mann–Whitney test. Four groups were compared with each other. *p<0.05, **p<0.01, ***p<0.001, ****p<0.0001.



 **Figure S4 Plasma from ACLF promotes macrophage M1 transformation.**

(a-b) Peripheral monocytes from HC were differentiated into macrophage in vitro by the addition of M-CSF for 7 days. Differentiated macrophages from HC were incubated in 20% ACLF plasma or HC plasma for 24 hours in the presence of (a) LPS (100 ng/ml) or (b) IL-4 (15 ng/ml). M1 phenotypes were assessed by the expression of CD80, CD86 and HLA-DR, and M2 phenotypes were assessed by the expression of CD206 and CD163.

(c-d) PMA-differentiated THP-1 was incubated in 20% ACLF plasma or HC plasma for 24 hours in the presence of (c) LPS (100 ng/ml) or (d) IL-4 (15 ng/ml). M1 phenotypes were assessed by the expression of CD86, and M2 phenotypes were assessed by PD-1.

All experiments were analyzed by flow cytometry. The horizontal line represented the mean. Statistical analyses were performed using the Mann–Whitney test. *p<0.05, **p<0.01, ***p<0.001. ns, not statistically significant.





**Figure S5 EP2 expression on blood immune cells.** (a) CD8^+^T cells. (b) CD4+T cells. (c) NK cells. (d) Monocyte. (e) Neutrophils. (f) NKT cells. The horizontal line represented the median, and statistical analyses were performed using the Mann–Whitney test. *p<0.05, **p<0.01. ns, not statistically significant.



 **Figure S6 Frequency of CXCR3^+^ CD8^+^ T cells and CXCR3^+^ monocytes.** The horizontal line represented the median. Statistical analyses were analyzed using the Mann–Whitney test. *p<0.05, **p<0.01. ns, not statistically significant.





**Figure S7 Association of PGE2-EP2 expression with disease severity in ACLF.**

(a) EP2 expression on CD8^+^ T cells in survivors or death or transplantation group during the 90-day follow-up.

(b) Plasma PGE2 levels were measured in survivors or death or transplantation group during the 90-day follow-up.

(c) Plasma PGE2 levels were measured in ACLF or ACLF-MOF during the 90-day follow-up.

(d) Correlation analysis between the plasma PGE2 levels and prognostic score.

The horizontal line represented the median. Statistical analyses were analyzed using the Mann–Whitney test or spearman correlation. ns, not statistically significant.
